# The impact of universal access to direct-acting antiviral therapy on the hepatitis C cascade of care among individuals attending primary and community health services

**DOI:** 10.1371/journal.pone.0235445

**Published:** 2020-06-30

**Authors:** Michael W. Traeger, Alisa E. Pedrana, Daniela K. van Santen, Joseph S. Doyle, Jessica Howell, Alexander J. Thompson, Carol El-Hayek, Jason Asselin, Victoria Polkinghorne, Dean Membrey, Fran Bramwell, Allison Carter, Rebecca Guy, Mark A. Stoové, Margaret E. Hellard, on behalf of the EC Victoria Partnership and the Australian Collaboration for Coordinated Enhanced Sentinel Surveillance of Blood-borne Viruses and Sexually Transmitted Infections (ACCESS)

**Affiliations:** 1 Burnet Institute, Melbourne, Victoria, Australia; 2 School of Public Health and Preventive Medicine, Monash University, Melbourne, Victoria, Australia; 3 Department of Infectious Diseases, Alfred Health and Monash University, Melbourne, Victoria, Australia; 4 Department of Gastroenterology, St Vincent’s Hospital, Melbourne, Victoria, Australia; 5 Cohealth, General Practice, Melbourne, Victoria, Australia; 6 Kirby Institute, University of New South Wales, Sydney, New South Wales, Australia; 7 School of Psychology and Public Health, La Trobe University, Melbourne, Australia; Centers for Disease Control and Prevention, UNITED STATES

## Abstract

**Background:**

Hepatitis C elimination will require widespread access to treatment and responses at the health-service level to increase testing among populations at risk. We explored changes in hepatitis C testing and the cascade of care before and after the introduction of direct-acting antiviral treatments in Victoria, Australia.

**Methods:**

De-identified clinical data were retrospectively extracted from eighteen primary care clinics providing services targeted towards people who inject drugs. We explored hepatitis C testing within three-year periods immediately prior to (pre-DAA period) and following (post-DAA period) universal access to DAA treatments on 1^st^ March 2016. Among ever RNA-positive individuals, we constructed two care cascades at the end of the pre-DAA and post-DAA periods.

**Results:**

The number of individuals HCV-tested was 13,784 (12.2% of those with a consultation) in the pre-DAA period and 14,507 (10.4% of those with a consultation) in the post-DAA period. The pre-DAA care cascade included 2,515 RNA-positive individuals; 1,977 (78.6%) were HCV viral load/genotype tested; 19 (0.8%) were prescribed treatment; and 12 had evidence of cure (0.5% of those RNA-positive and 63.6% of those eligible for cure). The post-DAA care cascade included 3,713 RNA-positive individuals; 3,276 (88.2%) were HCV viral load/genotype tested; 1,674 (45.1%) were prescribed treatment; and 863 had evidence of cure (23.2% of those RNA-positive and 94.9% of those eligible for cure).

**Conclusion:**

Marked improvements in the cascade of hepatitis C care among patients attending primary care clinics were observed following the universal access of DAA treatments in Australia, although improvements in testing were less pronounced.

## Introduction

Hepatitis C virus infection remains a major contributor to morbidity and mortality worldwide, with an estimated 400,000 deaths attributable to hepatitis C annually. [1] Following the advent of highly effective and tolerable direct-acting antiviral (DAA) treatments for hepatitis C infection, the World Health Organization (WHO) set global elimination targets, calling for an 80% reduction in incidence of chronic hepatitis C infection and a 65% reduction in annual hepatitis C related deaths by 2030 from 2015 levels. [1] Realisation of such targets requires widespread access to diagnostic testing and treatment for people living with hepatitis C, with incidence reductions particularly dependent on services reaching people who inject drugs (PWID), alongside high coverage of harm reduction services and needle and syringe programs. [2,3]

Australia has a long standing strategic response to hepatitis C, with the first National Hepatitis C Strategy released in 2000 [4] and ongoing national targets underpinning Australia’s response. A major catalyst in Australia’s response to hepatitis C was the decision to make DAA treatments available through the national Pharmaceutical Benefits Scheme (PBS) in 2016. [5,6] The PBS is a publically funded scheme which provides highly subsidised prescription drugs to individuals who qualify for Australia’s universal healthcare system. Australia became one of the first countries to make DAA prescriptions available regardless of treatment history or drug use status, and to allow treatment to be initiated by general practitioners and credentialed nurse practitioners outside of the tertiary setting. [6]

At the time DAA treatments were listed on the PBS in March 2016, there were an estimated 230,000 Australians living with chronic hepatitis C, [7] and up to December 2018, more than 74,000 people were estimated to have received DAA treatment for hepatitis C. [8] However, the monthly number of people treated has steadily declined over this time, with an average less than 1,500 people treated per month between July and December 2018. [8,9] While Australia has been identified as one of the few countries on track to meet WHO hepatitis C elimination targets, [10,11] the decline in numbers of people treated for hepatitis C in Australia is concerning. Hepatitis C elimination models show that maintaining high rates of hepatitis C testing and treatment among PWID, combined with maintaining a high quality and coverage of harm reduction programs, is essential to achieve elimination in Australia. [12] Monitoring of population-level hepatitis C testing and treatment and the progression of PWID through the hepatitis C cascade of care is therefore vital to assess progress towards hepatitis C elimination and identify gaps in service access to inform appropriate responses.

*The Australian Collaboration for Coordinated Enhanced Sentinel Surveillance of Blood-borne Viruses and Sexually Transmitted Infections* (ACCESS) sentinel surveillance system monitors sexually transmitted infection (STI) and blood-borne virus (BBV) testing and treatment outcomes among key populations. ACCESS data enables linkage of individuals’ episodes of care over time and across services, allowing for analysis of hepatitis C cascades of care among individuals with chronic hepatitis C infection at multiple time points. We used ACCESS data to explore changes in hepatitis C testing and the cascade of care among individuals with hepatitis C infection before and after universal access of DAAs in the state of Victoria, Australia.

## Methods

### Data extraction and study population

The features of the ACCESS surveillance systems have been published elsewhere. [13] Briefly, deidentified patient data are routinely extracted from participating services using specialised software called GRHANITE^™^, which creates an anonymous unique identifier from patient details. This identifier allows anonymous linkage between and within participating sites, creating a national cohort to facilitate epidemiological monitoring and the evaluation of clinical and public health interventions. For this analysis, clinical data were extracted from 18 services participating in ACCESS in the state of Victoria, Australia, including ten community health centres, seven primary care general practice clinics, and one specialist drug and alcohol service. Included clinics were sentinel surveillance sites selected based on high hepatitis C caseloads and provision of services targeting PWID, including opioid substitution therapy (OST) prescribing and co-location with needle and syringe programs. Patient demographics, hepatitis C pathology results (antibody, quantitative and qualitative RNA and genotype tests) and hepatitis C treatment data (treatment regimen, prescription date and treatment length) from January 1^st^ 2011 up to 28^th^ February 2019 were retrospectively extracted using GRHANITE^™^ data extraction software, which was designed specifically for the secure collection of deidentified health data. Patient records are linked within and across sites using a highly sensitive algorithm which utilises non-identifying probabilistic linkage keys derived from, but not containing, patient identifiers, including patient name, date of birth, sex and Medicare card number. [14]

### Hepatitis C testing before and after DAAs

We explored hepatitis C testing across the ACCESS network within the three-year periods immediately prior to (pre-DAA period) and following (post-DAA period) universal access to DAA treatments in Australia on the 1^st^ March 2016. We calculated the number and proportion of individuals attending a participating clinic who had a recorded hepatitis C antibody and/or RNA test, as well as the total number of hepatitis C tests conducted across the clinical network, within each three-year period (pre-DAA and post-DAA). We calculated the number of individuals with a positive antibody and RNA test result recorded within each three-year period, respectively. We also calculated the number of individuals attending a participating clinic within each period who had evidence of ever being HCV antibody tested since 1^st^ January 2011. As potentially identifying data stored in the patient notes are not extracted, it was not possible to determine the proportion of individuals attending the clinics who were people who inject drugs. To account for this, we explored hepatitis C testing and positivity among individuals with evidence of being prescribed OST (since January 2001) as a marker of injecting drug use and hepatitis C risk. [15]

### Hepatitis C care cascades

To explore changes in linkage to care and within-clinic treatment uptake among patients diagnosed with hepatitis C, we then constructed hepatitis C cascades of care among individuals who had evidence of ever being diagnosed hepatitis C RNA-positive. We constructed two cascades, each reflecting the status of individuals at two time points representing the end of the pre-DAA and post-DAA study periods; 28^th^ February 2016 (pre-DAA cascade) and 28^th^ February 2019 (post-DAA cascade). Cascades were restricted to individuals who had a clinical consultation within the respective three-year study period. Individuals were included in both cascades if they had a consultation in both periods.

For the cascades analyses, retrospective data from 1^st^ January 2011 was used to classify individuals as ever diagnosed RNA-positive, i.e. individuals with any positive RNA test result between 1^st^ Jan 2011 and 28^th^ February 2016 (pre-DAA cascade) or between 1^st^ Jan 2011 and 28^th^ February 2019 (post-DAA cascade) were included in the cascade analyses. Individuals were categorised as having reached one of four cascade stages at the end of the respective period; (1) diagnosed RNA-positive; (2) tested for HCV viral load or HCV genotype; (3) prescribed hepatitis C treatment within the clinical network; and (4) having evidence of cure. Cascade stages were adapted from the recently developed international Consensus Hepatitis C Cascade of Care, [16] with ‘tested for HCV viral load or HCV genotype’ reflecting linkage to hepatitis C care, a supplementary stage included in the Consensus Cascade. Individuals with an electronic prescription for hepatitis C treatment were assumed to be diagnosed RNA-positive and were included in cascade, even if no positive RNA test result or HCV viral/genotype test was recorded in patient management systems. Individuals with a negative RNA test result at least 48 weeks (interferon-based treatments) or 8 weeks (DAA treatments) after the date of treatment initiation were classified as having evidence of cure. While current Australian guidelines for hepatitis C treatment recommend post-treatment monitoring in the form of an RNA test twelve weeks post-treatment-completion, [17] previous guidelines also recommended on-treatment monitoring at four weeks post-treatment initiation and at treatment completion. [18] However, many of the treated individuals in our network did not return for SVR testing twelve weeks post-treatment completion. Given that the shortest prescribed treatment length was eight weeks, and the cure rate of DAA treatments is above 95% [19], we classified those with an RNA negative result eight weeks or more post-treatment initiation as having evidence of cure in the post-DAA period.

To explore and account for rates of return RNA testing post-treatment, we calculated the number of individuals eligible for progressing to the final stage (having evidence of cure), i.e. the number of individuals with a determinate RNA test result at least 48 weeks (interferon-based treatments) or 8 week (DAA treatments) post-treatment-initiation. We then calculated the proportion of these individuals whose RNA test result post-treatment-initiation was negative.

As treatment initiation was inferred by presence of an electronic prescription for hepatitis C treatment stored in patient management systems of participating clinics, and as some individuals were likely prescribed treatment at clinics not participating in the ACCESS project, we calculated the number of individuals who did not have a matched electronic prescription in the ACCESS database but subsequently had evidence of RNA clearance. This was calculated as the number of RNA-positive individuals with no electronic prescription recorded at a participating clinic who had a subsequent negative RNA test result at least 48 weeks (in the pre-DAA period) or 8 weeks (in the post-DAA period) after their last positive RNA test result. These individuals may represent individuals treated at a participating clinic and achieving cure, but with no electronic prescription recorded, or individuals treated outside of the clinical network returning to a participating clinic for post-treatment RNA monitoring.

Given the defined length of time between treatment initiation and date of negative RNA test result required to be classified as having evidence of cure in our cascade (48 weeks for interferon-based treatment and 8 weeks for DAA treatments), it is possible that some individuals initiated treatment too late to be eligible for the cure stage. To explore any impact this may have on our cascades, we calculated the proportion of individuals initiating treatment in the pre-DAA and post-DAA periods less than 48 weeks or 8 weeks prior to the cascade observation points, respectively.

### Ethics approval

Ethics approval for the ACCESS project in Victoria was provided by the Alfred Hospital Human Research Ethics Committee (Project 248/17), as well as several specialised committees for key populations, including ACON, Thorne Harbour Health, and the Aboriginal Health and Medical Research Council. As our study analyses de-identified data collected under the auspices of public-health surveillance, individual patient consent was not required. Individuals were able to opt out of the ACCESS surveillance system if they wish.

## Results

### Participants

The number of individuals with a clinical consultation at a participating ACCESS clinic at least once in the pre-DAA and post-DAA periods was 93,856 and 113,832, respectively; 51,562 individuals had a consultation in both periods, making for a total of 156,126 individuals contributing to our analyses. Those visiting a clinic in the pre-DAA and post-DAA periods were 50.8% and 51.7% female, respectively, and the mean age was 43 across both periods. In the pre-DAA period, 10,546 (11.2%) individuals had evidence of OST prescription, and in the post-DAA period 11,061 (9.7%) individuals had evidence of OST prescription (Table 1).

**Table 1:** Age and sex of individuals attending a participating ACCESS clinic during the study period.

	Pre-DAA cohort—Attended a clinic	Post-DAA cohort—Attended
Total*	93,856	113,832
Sex, n (%)		
Female	47,676 (50.8)	58,850 (51.7)
Male	44,957 (47.9)	53,422 (46.9)
Other	499 (0.5)	938 (0.8)
Unknown/missing	724 (0.8)	622 (0.6)
Age^, n (%)		
18–19	2,242 (2.4)	2,464 (2.2)
20–29	20,885 (22.3)	24,638 (21.6)
30–39	23,574 (25.1)	28,807 (25.3)
40–49	17,678 (18.8)	21,258 (18.7)
50+	29,477 (31.4)	36,665 (32.2)
Mean age	43.0	43.4
History of OST prescription#	10,546 (11.2)	11,061 (9.7)

*51,562 individuals were had a visit during each period and were included in both cohorts

^Age at end of 2016 for the pre-DAA cohort and age at end of 2019 for the post-DAA cohort

^#^ Any prescription of Methadone, Suboxone, Buprenorphine or Naltrexone since Jan 1^st^ 2011.

### Hepatitis C testing

The number of individuals tested for hepatitis C (antibodies and/or RNA) at least once within the three-year pre-DAA period was 13,784 (14.7% of those who had a consultation); of which 1,822 individuals (13.7%) had positive antibody test result and 1,918 individuals (13.9%) had a positive RNA test result recorded during the pre-DAA study period. During the pre-DAA study period, a total of 14,267 antibody tests and 3,636 RNA tests were performed. Among those with evidence of OST prescription, 3,044 (28.9%) were tested for hepatitis C (antibodies and/or RNA) during the pre-DAA period (Table 2).

**Table 2:** Number of individuals hepatitis C tested and number positive in the pre-DAA and post-DAA periods.

	Pre-DAA period– 1^st^ March 2013—28^th^ February 2016		Post-DAA period– 1^st^ March 2016—28^th^ February 2019	
	All individuals	Individuals with evidence of OST^#^	All individuals	Individuals with evidence of OST^#^
Number of individuals who had a consultation at a participating ACCESS clinic	93,856	10,546	113,832	11,061
Number of hepatitis C antibody tests performed within three-year study period	14,267	2,434	14,236	2,697
Number of hepatitis C RNA tests performed within three-year study period	3,636	2642	6,771	5,276
Number of individuals antibody and/or RNA tested within three-year study period (% of those who had a consultation)	13,784 (14.7)	3,044 (28.9%)	14,507 (12.7)	4,042 (36.5%)
Number of individuals antibody tested within three-year study period (% of those who had a consultation)	12,491 (13.3)	2,063 (19.6%)	12,347 (10.8)	2,342 (21.2%)
Number of individuals RNA tested within three-year study period (% of those who had a consultation)	2,745 (2.9)	2,018 (19.1%)	4,325 (3.8)	3,313 (30.0%)
Number of individuals antibody positive within three-year study period (% of those antibody tested)	1,822 (15.1)	1,322 (64.1%)	2,070 (16.8)	1,546 (66.0%)
Number of individuals RNA positive within three-year study period (% of those RNA tested)	1,918 (69.9)	1,465 (72.6%)	2,419 (55.9)	1,946 (58.7%)
Number of individuals ever tested for antibody and/or RNA since 1^st^ January 2011 (% of those who had a consultation)	17,285 (18.4)	4,232 (40.1%)	23,782 (20.9)	5,770 (52.2%)

^#^ Any prescription of Methadone, Suboxone, Buprenorphine or Naltrexone since Jan 1^st^ 2011.

The number of individuals tested for hepatitis C (antibodies and/or RNA) at least once within the three-year post-DAA study period was 14,507 (12.7% of those who had a consultation); of which 2,070 individuals (14.3%) had positive antibody test result and 2,419 individuals (16.7%) had a positive RNA test result recorded during the post-DAA study period. During the post-DAA study period, a total of 14,236 antibody tests and 6,771 RNA tests were performed. Among those with evidence of OST prescription, 4,042 (36.5%) were tested for hepatitis C (antibodies and/or RNA) during the post-DAA period (Table 2).

The proportion of individuals with a clinical consultation in each respective period who had recorded evidence of ever being tested for hepatitis C (antibody and/or RNA) since January 2011 slightly increased from 18.4% at the end of the pre-DAA period (17,285/93,856) to 20.9% at the end of the post-DAA period (23,782/113,832). Among those with evidence of OST, the proportion ever hepatitis C tested increased from 40.1% at the end of the pre-DAA period (4,232/10,546) to 52.2% at the end of the post-DAA period (5,770/11,061) (Table 2).

### Cascades of care among RNA-diagnosed individuals

#### Pre-DAA cascade

**Fig 1:**
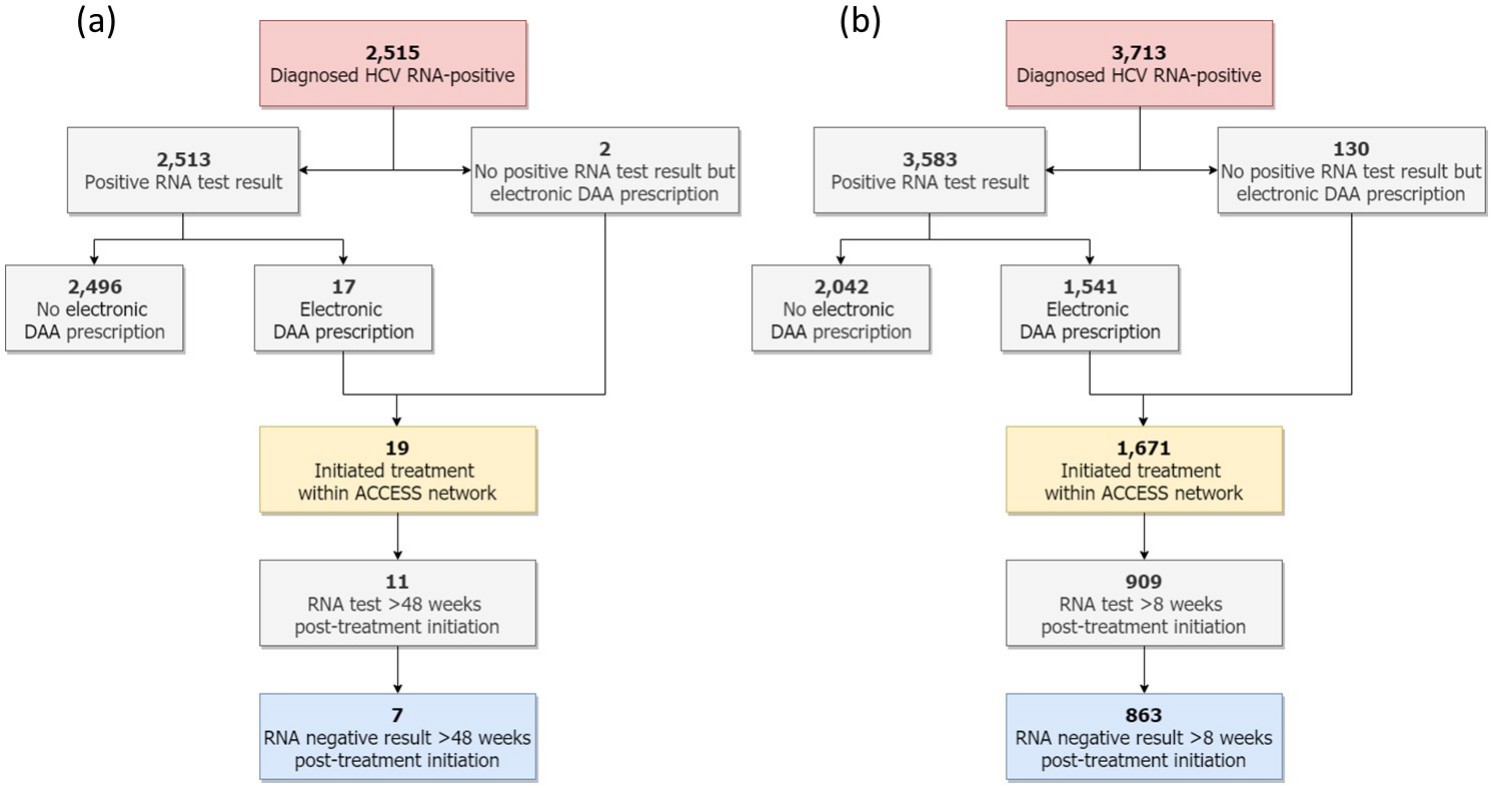
Flow diagram showing treatment and cure outcomes among individuals classified as ever diagnosed RNA-positive with a consultation in the (a) pre-DAA period and (b) post-DAA period.

**Fig 2:**
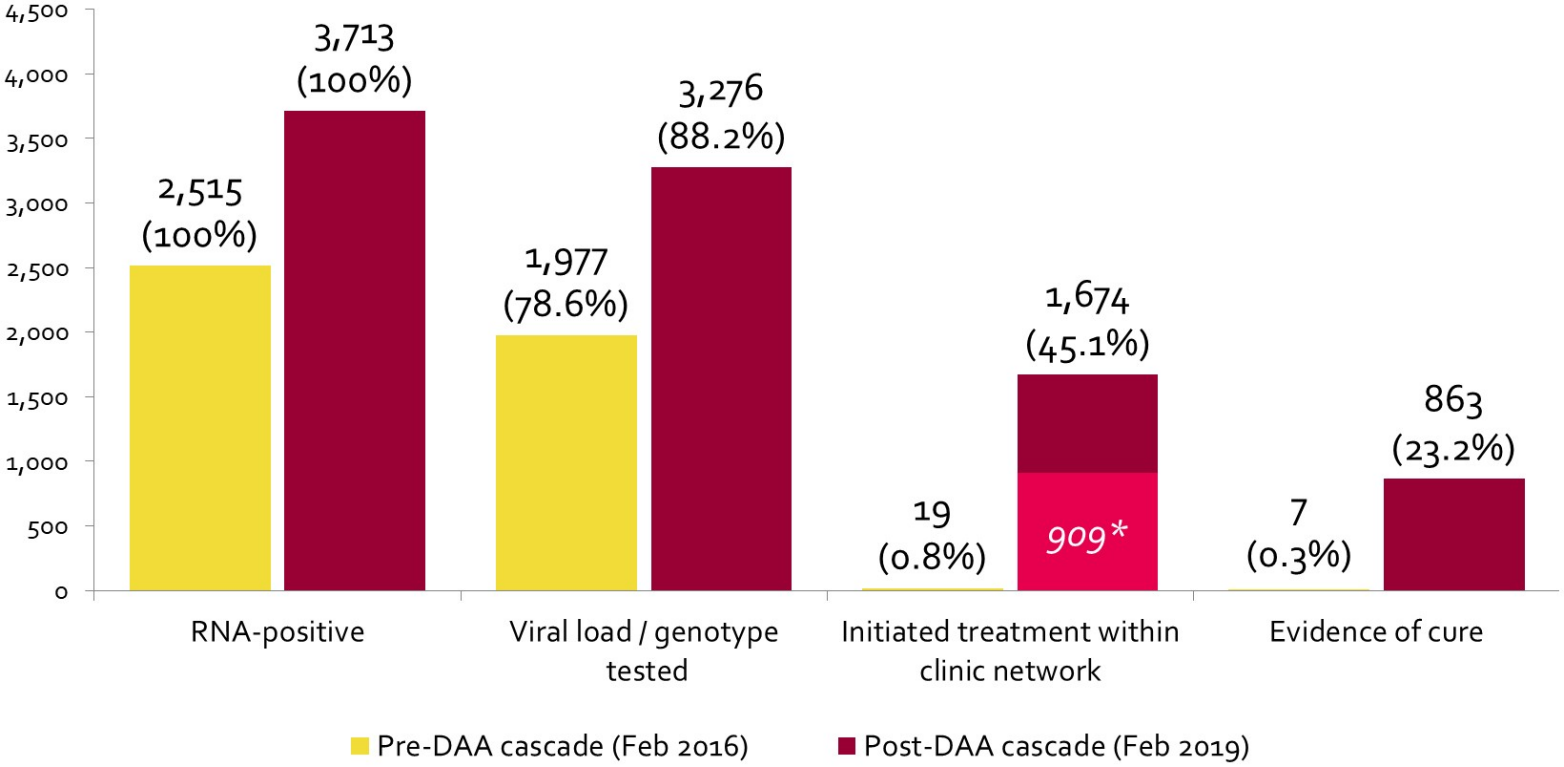
Number of individuals visiting an ACCESS clinic with the pre-DAA and post-DAA study periods classified as ever diagnosed RNA-positive in each stage of care at the end of the pre-DAA period and the post-DAA period. Percentages represent proportion of individuals with evidence of ever being diagnosed RNA positive prior to each cascade. *Light-shaded section represents individuals in the post-DAA cascade who had a recorded RNA test at least 8 weeks post-treatment-initiation and were eligible to progress to the having ‘evidence of cure’ stage (n = 909). 11 of the 19 individuals who initiated treatment in the pre-DAA cascade had a recorded RNA test at least 48 weeks post-treatment-initiation and were eligible to progress to having evidence of cure.

Among individuals with a clinical consultation in the pre-DAA period, 2,515 were classified as ever diagnosed RNA-positive prior to 28^th^ February 2016 and were included in the pre-DAA cascade (1,918 had a recorded positive RNA test result during the pre-DAA study period, 595 had a recorded positive RNA test result prior to the pre-DAA study period and two had an electronic prescription for hepatitis C treatment but no positive RNA test result) (Fig 1a). Of these 2,515 RNA-positive individuals, 70.2% were male and the mean age was 43.6 years (Table 3). At the end of the pre-DAA period, 1,977 (78.6%) had evidence of a viral load / genotype test. Of the 2,513 individuals with a recorded positive RNA test result, 17 had an electronic prescription for hepatitis C treatment, making for a total of 19 individuals (0.8% of those diagnosed RNA-positive) classified as having initiated hepatitis C treatment. Of these, 11 (57.9%) had an RNA test at least 48 weeks post-treatment-initiation; seven individuals (0.3% of those diagnosed RNA-positive and 63.6% of those eligible for having evidence of cure) had a negative RNA test result at least 48 weeks post-treatment initiation and were classified as having evidence of cure. (Fig 2).

**Table 3:** Age and sex of individuals with evidence of ever being RNA positive and included in cascades of care.

	Pre-DAA cohort—Ever RNA positive	Post-DAA cohort—Ever RNA positive
Total*	2,515	3,713
Sex, n (%)		
Female	746 (29.7)	1134 (30.5)
Male	1765 (70.2)	2572 (69.3)
Other	4 (0.2)	6 (0.2)
Unknown/missing	0 (0.0)	1 (0.0)
Age^, n (%)		
18–19	4 (0.2)	0 (0.0)
20–29	123 (4.9)	117 (3.2)
30–39	811 (32.2)	890 (24.0)
40–49	902 (35.9)	1511 (40.7)
50+	675 (26.8)	1195 (32.2)
Mean age	43.6	45.7
History of OST prescription#	1,961 (78.0)	2,966 (79.9)

*51,562 individuals were had a visit during each period and were included in both cohorts

^Age at end of 2016 for the pre-DAA cohort and age at end of 2019 for the post-DAA cohort

^#^ Any prescription of Methadone, Suboxone, Buprenorphine or Naltrexone since Jan 1^st^ 2011.

#### Post-DAA cascade

Among individuals with a clinical consultation in the post-DAA period, 3,713 individuals were classified as ever diagnosed RNA-positive prior to 28^th^ February 2019 and were included in the post-DAA cascade (2,419 had a recorded positive RNA result during the post-DAA study period, 1,164 had a recorded positive RNA test result prior to the post-DAA study period and 130 had an electronic prescription for hepatitis C treatment but no positive RNA test result) (Fig 1a). Of these 3,713 RNA-positive individuals, 69.3% were male and the mean age was 45.7 years (Table 3). At the end of the post-DAA period, 3,276 (88.2%) had evidence of a viral load / genotype test. Of the 3,583 individuals with a recorded positive RNA test result, 1,541 had an electronic prescription for hepatitis C treatment, making for a total of 1,671 individuals (45.1% of those diagnosed RNA-positive) classified as having initiated hepatitis C treatment. Of those, 909 (54.4%) had an RNA test at least 8 weeks post-treatment-initiation; 863 individuals (23.2% of those diagnosed RNA-positive and 94.9% of those eligible for having evidence of cure) had a negative RNA test result at least 8 weeks post-treatment initiation and were classified as having evidence of cure (Fig 2).

Among the 2,496 individuals classified as diagnosed RNA-positive included in the pre-DAA cohort and with no electronic prescription for hepatitis C treatment recorded, 76 had a subsequent negative RNA test result at a participating clinic at least 48 weeks after their last RNA-positive test, which may be indicative of treatment monitoring and cure. Among the 2,042 individuals classified as diagnosed RNA-positive included in the post-DAA cohort and with no electronic prescription for hepatitis C treatment recorded, 477 (23.5%) had a subsequent negative RNA test result at a participating clinic least 8 weeks after their last RNA-positive test, which may be indicative of treatment monitoring and cure (Fig 2).

Of the 19 individuals classified as treated in the pre-DAA era, all were prescribed treatment at least 48 weeks prior to the end of the cascade period, 28^th^ February 2016. Of the 1,671 individuals classified as treated in the pre-DAA era, 1,635 (97.8%) were prescribed treatment at least 8 weeks prior to the end of the cascade period, 28^th^ February 2019. As such, date of treatment initiation prior to cascade cut off dates would have little effect on our cascades.

## Discussion

In this sentinel surveillance network of community health centres and general practices providing clinical services targeted towards people who inject drugs, we observed a marked improvement in the hepatitis C cascade of care following the introduction of widely-available DAA treatments for hepatitis C in Australia. The proportion of RNA-positive individuals initiating treatment and with recorded evidence of cure improved substantially following DAA availability, however these improvements were contrasted by considerably less change in the overall hepatitis C testing rate between periods, which remained low.

The proportion of individuals ever diagnosed with hepatitis C who had evidence of initiating treatment within our primary care network increased from less than 1% to 45%. However, the proportion of patients visiting a clinic during each three-year period who had ever been tested for hepatitis C only slightly increased from 18.4% to 20.9%, and the overall proportion of individuals attending who were tested for hepatitis C during each respective period slightly decreased (14.7% to 12.7%); the number of antibody tests performed remained very stable at around 13,250 in each three-year period. Notably, among a subset of participants with a history of OST prescription, the proportion receiving hepatitis C testing increased from 28.9% to 36.5% across periods. While this suggests some improvements in risk-based testing, the increase in the proportion of OST recipients receiving hepatitis C testing was modest, and testing remained suboptimal across the entire study period, with approximately half of individuals with a history of OST who attended a clinic in the post-DAA period having no evidence of ever being hepatitis C tested within the ACCESS network. Overall, our data suggest that while the absolute number of individuals receiving treatment for chronic hepatitis C within this network of primary care and community health clinics increased dramatically, challenges remain regarding how to increase the number of patients accessing initial testing with subsequent linkage to treatment to optimal levels. A recent modelling study which utilises national-level testing data suggests that significant increases in hepatitis C testing are required in order to achieve elimination in Australia. [20]

The observed increase in hepatitis C treatment rates reflects widespread treatment of patients chronically infected with HCV following the listing of DAAs on the PBS, and may also be reflective of shifting of treatment from tertiary health settings to primary care and community health clinics. Prior to 2016, hepatitis C treatment prescribing was restricted to GPs or nurses with specialised training and standard practice was to refer patients to specialist care for treatment, and thus patients’ prescription data would not be captured. However, treatment initiation rates estimated in our pre-DAA cascade are reflected by low rates of treatment uptake in Australia reported prior to 2016, both nationally [9] and among PWID. [21] It is also likely that prior to the introduction of DAAs, clinicians were refraining from prescribing treatment in anticipation of new, more effective treatment regimens.

While we observed a marked improvement in treatment initiation in the primary care setting following DAA availability, the current cascade of care is far from perfect with less than 50% of those with an RNA-positive test result at their attending clinic having evidence of treatment prescription within the network by March 2019. Despite significant improvements in treatment efficacy and minimal side-effects of DAA treatments, barriers to treatment uptake remain in the DAA era. Qualitative research findings suggest limited knowledge of symptoms of hepatitis C and new treatment options among PWID, as well as considerable clinician/provider barriers to treatment initiation [22]. Evidence also suggests large variations in treatment uptake across geographical areas in Australia, driven by population characteristics, health service access and disease burden. [23]

Similar improvements in the hepatitis C cascade of care have been observed in other settings. Analysis of population-level hepatitis C testing data in British Colombia, Canada showed an increase in the proportion of RNA-positive individuals initiating treatment from 29% in 2012 to 55% in 2018, following the availability of DAA treatments in early 2015, and unrestricted access in April 2018. [24] Smaller community-clinic based studies have also shown similar rates of treatment uptake in the DAA era in the United States. [25]

Despite current Australian hepatitis C treatment guidelines recommending assessment of sustained virologic response at 12 weeks post-treatment (SVR12) through RNA testing, less than 55% of patients treated in our post-DAA cohort returned to a participating clinic for a subsequent RNA test at least eight weeks post-treatment-initiation. While post-treatment RNA monitoring was necessary for interferon-based treatment regimens, the high-efficacy of DAA treatments make post-treatment monitoring less important. Among those who returned to a participating clinic for RNA testing at post-treatment, the proportion of treated individuals who have evidence of cure in our analysis increased from 64% to 95% across periods. Given DAA efficacy has been shown to be above 95% in multiple populations and settings, [19] the level of effort that community services put into following up patients for SVR12 could equally or alternatively be put into following up patients that have not yet initiated treatment or testing those with ongoing risk of reinfection. There is limited real-world data to indicate why treated individuals are not returning for post-treatment monitoring, and whether treated individuals with ongoing risk for reinfection are regularly monitored through RNA testing.

While the types of clinics included in this sentinel surveillance project see patients for a range of health needs, many are co-located with needle and syringe exchange programs, and most have OST prescribers, and therefore see a large number of PWID. The proportion of patients attending these clinics who were screened for hepatitis C was less than 15% in both periods, and the proportion with evidence of ever being screened within the network was approximately 20%. While the low rate of testing may be indicative of targeted screening among a heterogeneous clinical population, the network-wide antibody-positivity rate (greater than 15% in both periods) highlights a significant opportunity to increase testing among these populations. Even among a subgroup of individuals with evidence of OST, whose antibody-positivity rate was greater than 65%, less than 40% received a hepatitis C test during either period. Increased screening will be necessary to identify individuals chronically infected with hepatitis C and to maintain treatment rates, and universal screening in settings with a high positivity rate will likely yield high numbers of new diagnoses. Routinely collected sentinel surveillance data, such as that used in this study, will play an important role in monitoring Australia’s progress towards hepatitis C elimination.

### Limitations

There are several limitations of our analyses. First, behavioural data including injecting drug use is not readily recorded in patient management systems of participating clinics. Therefore, we could not guarantee that all individuals included in the analysis were PWID. However, participating clinics are selected based on the provision of services targeting PWID, including OST prescribing and co-location with needle and syringe programs, and are expected to see a high caseload of PWID. We were also able to explore testing outcomes among individuals with a history of OST, a proxy for injecting drug use. Nevertheless, our findings may not be generalisable to the wider population of PWID in Australia. Second, as we relied on electronic prescriptions stored in patient management systems of participating clinics to define treatment initiation, it is noted that many individuals may have been treated outside of our clinical network, or in some cases may have been prescribed treatment via non-electronic handwritten scripts. However, using longitudinally linked data we were able to identify participants diagnosed with hepatitis C who subsequently returned to a participating clinic and tested RNA-negative, indicative of treatment. It is also possible that individuals were tested for hepatitis C outside of our clinical network. Third, we did not assess changes in time spent between cascade stages, rather, we compared cross-sectional cascades at two specific points in time. Fourth, we did not account for spontaneous clearance in our analyses, which may have led to an overestimate in the proportion of treated individuals achieving cure. Fifth, improvements in the cascade of care across periods may be, in part, due to individuals in the post-DAA cohort being more likely to initiate treatment given the longer duration of their hepatitis C infection, as individuals could be carried forward from the pre-DAA to post-DAA cohort. We also made an assumption that a negative RNA test at least eight weeks post-treatment initiation was indicative of being cured; as many individuals did not have an SVR12 test performed, we may have overestimated the number of individuals achieving cure in the post-DAA period. Finally, while we have explored a clinic network-level cascade of care, limited sociodemographic data precluded disaggregated analysis and identification of factors associated with progressing through the cascade of care.

## Conclusion

Marked improvements in the cascade of hepatitis C care among patients attending primary care and community health clinics were observed following the universal access of DAA treatments in Australia, highlighting the potential for other countries aiming for elimination to successfully integrate hepatitis C treatment into the community services. Despite improvements however, treatment uptake remains suboptimal, and rates of diagnostic testing did not significantly increase. Increased testing among populations at risk for hepatitis C will be necessary to identify individuals chronically infected with hepatitis C, maintain adequate levels of treatment uptake and achieve hepatitis C elimination at the national and global level.

## Abbreviations

ACCESS: Australian Collaboration for Coordinated Enhanced Sentinel Surveillance of Blood-borne Viruses and Sexually Transmitted Infections
BBV: Blood-borne virus
DAA: Direct-acting antiviral
HCV: Hepatitis C virus
OST: Opioid substitution therapy
PBS: Pharmaceutical Benefits Scheme
PWID: People who inject drugs
RNA: Ribonucleic acid
STI: Sexually transmitted infection
WHO: World Health Organization
